# Using peer-assisted learning to teach and evaluate residents’ musculoskeletal skills

**DOI:** 10.3402/meo.v20.27255

**Published:** 2015-05-29

**Authors:** Johanna Martinez, Christina Harris, Cathy Jalali, Judy Tung, Robert Meyer

**Affiliations:** 1 New York Presbyterian-Weill Cornell Medical Center, New York, NY, USA; 2 VA Greater Los Angeles Healthcare System, David Geffen School of Medicine at UCLA, Los Angeles, CA, USA

**Keywords:** medical education, clinical skills, OSCE, peer learning, physical exam

## Abstract

Although direct observation and corrective feedback are established methods of increasing select aspects of residents’ musculoskeletal (MSK) clinical skills, the evaluation and management of patients with MSK complaints remains an underemphasized part of internal medicine training. This paper reports on the development of an innovative peer-assisted learning (PAL) model to teach five MSK areas (back, knee, shoulder, neck, or hip pain). Based on data from 42 participating interns and 44 senior residents from an urban US academic medical center, results from an objective structured clinical exam (OSCE) demonstrate gains in both knowledge and self-reported confidence in MSK skills. Moreover, subsequent focus group results reveal a strong preference for the PAL model. In conclusion, an educational module that utilizes the OSCE format holds much promise for teaching MSK skills to both intern and senior residents.

Presently, musculoskeletal (MSK) patient complaints account for 38% of primary care visits in the US and are expected to increase with time (1). In response, there has been a push toward increasing MSK education in medical schools (2). However, despite these ongoing efforts, students still report low confidence in the MSK examination, and perform poorly on MSK basic competency examinations (3) – a gap that persists into residency training (4, 5). In response to these deficits, both undergraduate and graduate training programs have experimented with didactic lectures (6), small group interactive sessions (7, 8), computer-assisted learning modules (9, 10), and use of patient educators (11) to improve MSK skills.

Although objective structured clinical exams (OSCEs) are commonly used to assess learners’ skill development, they are also effective educational tools (12). By incorporating opportunities for immediate observer feedback, a teaching OSCE allows trainees to learn from their mistakes in the moment. OSCEs, both as evaluative and educational tools, have been widely utilized in medical school training, with more than 75% of medical schools requiring a comprehensive OSCE prior to graduation (13). In contrast, residency programs – such as general internal medicine (IM) – typically use OSCEs less frequently (14).

Peer-assisted learning (PAL), a model where teacher and learner are at similar educational levels (i.e., at a shorter “cognitive distance”), may be a viable alternative (15). In this approach, learners experience a comparatively safe and comfortable environment whereby the teacher has an opportunity to enhance his or her teaching and feedback skills. Since PAL in MSK education is limited, especially in residency education (16–18), we describe in detail and report the feasibility and effectiveness of one such attempt.

## Methods

**Fig. 1:**
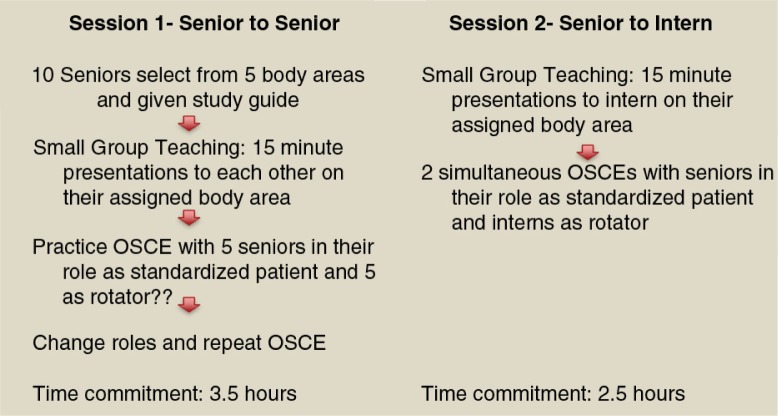
Layout of teaching sessions.

Participants were IM residents at New York Presbyterian-Weill Cornell Medical Center, a large academic program in New York City. Our MSK module was comprised of two consecutive sessions during the residents’ ambulatory block time, and included 43 and 45 PAL dyads of interns and senior residents, respectively (see Fig. 1). The intervention was repeated four times over 2 months to allow participation by all designated program trainees.

### Session 1: senior to senior teaching

**Fig. 2:**
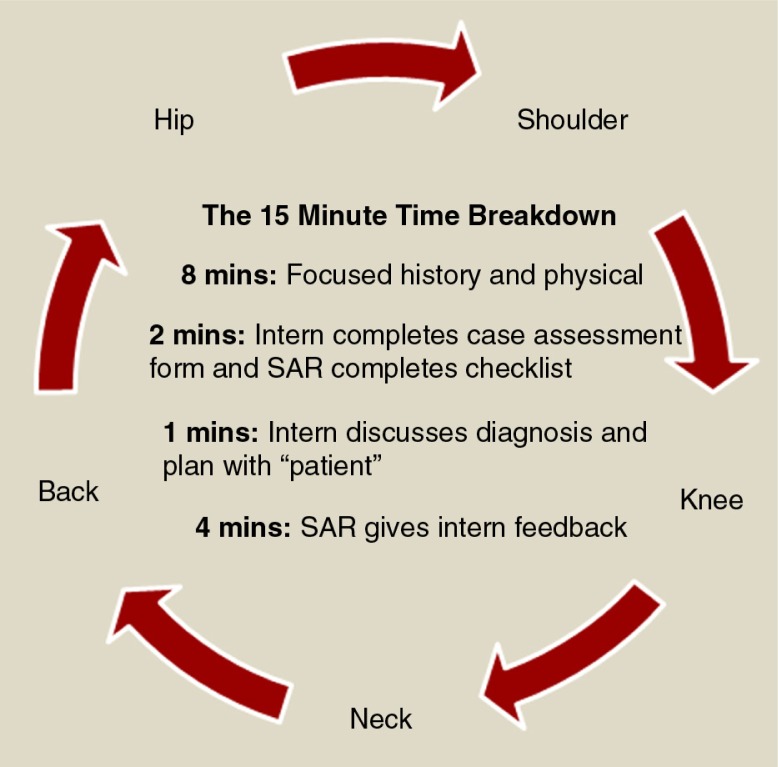
Time allotments for each of the five OSCE stations.

Prior to the first session, which focused on senior learning and training, each senior resident was provided with a study guide detailing the relevant anatomy, history, physical examination, differential diagnosis, and appropriate treatment plan for each of five common MSK complaints: back, knee, shoulder, neck, and hip pain. In groups of two to three, senior residents were instructed to learn and then deliver to their peers a 15-min interactive presentation (Fig. 2) on the assigned MSK scenario, which attending physicians observed. Immediately following the presentations, senior residents participated in practice OSCEs, during which they had the opportunity to serve as standardized patients (SPs) and practice providing corrective feedback on their assigned MSK joint. They could also rotate through the four additional stations as learners. Two faculty members were present to ensure completeness and accuracy of the senior residents’ techniques and provide appropriate, real-time feedback.

### Session 2: senior to intern teaching

In this session, interns first rotated through senior residents’ assigned 15-min presentations emphasizing appropriate techniques for each respective MSK physical exam (PE) (Fig. 2). Following this, interns rotated through a five-station OSCE where the senior residents served as SPs – who were expected to provide corrective feedback using an author-developed checklist. This study was approved by the Weill Cornell Medical College Institutional Review Board.

## Evaluation

Our primary goal was to examine the feasibility and acceptability of peer-assisted teaching of the MSK exam. Using a 5-point Likert scale, residents rated their self confidence in: 1) taking a history of present illness (HPI); 2) performing a PE; and 3) delivering a treatment plan (Tx) in patients with back, knee, shoulder, neck, and hip pain. Medical knowledge was assessed via a 15-item multiple-choice test developed by the authors.

For each MSK area, attitudinal/confidence scores were summed across HPI, PE, and Tx, resulting in scores ranging from 5 to 25. Paired and independent samples *t*-tests, along with a non-parametric alternative (Wilcoxon sums rank test), were used to compare mean within- and between-subjects differences. Finally, focus groups were conducted to qualitatively explore residents’ experience with this method of teaching. A critical *p*-value of <0.05 was established for all inferential analyses.

## Results

In total, 86 IM trainees (42 interns, 44 senior residents) participated in the OSCE – 73 of whom (85%) completed the pre-post survey and all parts of the PAL MSK curriculum. For various reasons, paired and non-paired data were available for 66 and 73 residents, respectively. Internal consistency of the 15-item knowledge assessment was acceptable (α = 0.65).

Not surprisingly, senior residents at baseline were significantly more confident than interns in all parts of the MSK evaluation: HPI (21.1 vs. 18.1), PE (18.0 vs. 15.4), and Tx (18.4 vs. 15.7). Trainees at both levels were significantly more confident in the former than either of the latter. Postintervention, senior residents were significant more confident in the PE and Tx arenas, whereas interns reported significant increases in all parts of the MSK evaluation. Increase in senior residents’ confidence did not vary according to assigned MSK area.

Despite differences in mean baseline confidence, no significant knowledge differences were found between interns and senior residents at either baseline (48 vs. 52%) or postintervention (74 vs. 69%). Both trainee groups showed significant (but comparable) increases in MSK-related knowledge over time.

Postintervention focus groups with 35 residents revealed that: 1) MSK content was seen as highly relevant and should be a structured part of the IM residency curriculum; 2) time dedicated to MSK education in medical school and residency is presently inadequate; and 3) interns preferred learning from senior residents rather than attendings. Additionally, senior residents embraced the opportunity to improve their teaching skills and identified attendings’ corrective feedback as the most effective component of their teaching preparation.

However, focus group comments also highlighted areas for improvement. Both intern and senior residents criticized the intensity of the intervention, and senior residents found teaching a particular topic five consecutive times in short time to be “unrealistic.” Interns, in contrast, felt overwhelmed with the sheer volume of information covered in the span of few hours and suggested that a longitudinal curriculum be developed to cover fewer joints in greater depth over more sessions.

## Discussion

In documenting our MSK module, which included a concise study guide, small group teaching, and a PAL OSCE, we found that the PAL model can enhance both learners’ and teachers’ MSK-related confidence and knowledge. Despite feeling unrealistic, repetition of the small group presentations and OSCE stations was intentional (19, 20) and fairly well-received. Senior residents experienced significant gains in confidence and medical knowledge, and were also able to refine their skills related to teamwork, small group facilitation, formative feedback, and remediation. Interns, in contrast, benefited from being taught by someone closer to their own educational level.

Previous attempts to utilize OSCEs to evaluate residents’ MSK competency have been few. Greisser and associates developed a 4-station OSCE for orthopedic residents focused on history-taking and physical examination skills (21), whereas Smith and colleagues developed a shorter, 2-station OSCE to evaluate residents’ examination skills of the knee and shoulder (7). Recognizing the assessment limitations of the OSCE (12, 13), and the uniqueness of graduate medical education, our primary use of this model was teaching. In this vein, comparable “teaching OSCE” was found to be better received by faculty and students with regards to educational impact and real-time feedback (12).

Our curricular evaluation has limitations – namely, the lack of a control group and a reliance on self-report. Furthermore, although residents’ confidence and medical knowledge improved significantly, we did not explicitly evaluate clinical skill. That is, our outcomes focused solely on perceived confidence and associated knowledge; we did not formally assess history-taking related to MSK (for example). Finally, we were not able to assess examiner reliability among the attendings and senior residents who observed each case.

In summary, our implementation of PAL-guided teaching OSCEs using senior residents as learners, evaluators, and educators may serve as a basic template increasing IM trainees’ MSK-related confidence and knowledge. Our next step in further refining this curriculum is to more rigorously examine actual skill development and retention over time.
